# Elevation of Cellular BPDE Uptake by Human Cells: A Possible Factor Contributing to Co-Carcinogenicity by Arsenite

**DOI:** 10.1289/ehp.9284

**Published:** 2006-08-18

**Authors:** Shengwen Shen, Jane Lee, Xuejun Sun, Hailin Wang, Michael Weinfeld, X. Chris Le

**Affiliations:** 1 Department of Public Health Sciences and Department of Laboratory Medicine and Pathology and; 2 Department of Oncology, Faculty of Medicine and Dentistry, University of Alberta, Edmonton, Canada

**Keywords:** arsenic, benzo[*a*]pyrene, cellular uptake, co-carcinogenicity, DNA adducts

## Abstract

**Background:**

Arsenite (iAs^III^) can promote mutagenicity and carcinogenicity of other carcinogens. Considerable attention has focused on interference with DNA repair by inorganic arsenic, especially the nucleotide excision repair (NER) pathway, whereas less is known about the effect of arsenic on the induction of DNA damage by other agents.

**Objectives:**

We examined how arsenic modulates DNA damage by other chemicals.

**Methods:**

We used an NER-deficient cell line to dissect DNA damage induction from DNA repair and to examine the effects of iAs^III^ on the formation of benzo[*a*]pyrene diol epoxide (BPDE)–DNA adducts.

**Results:**

We found that pretreatment with iAs^III^ at subtoxic concentrations (10 μM) led to enhanced formation of BPDE–DNA adducts. Reduced glutathione levels, glutathione *S-*transferase activity and chromatin accessibility were also measured after iAs^III^ treatment, but none of these factors appeared to account for the enhanced formation of DNA adducts. However, we found that pretreatment with iAs^III^ increased the cellular uptake of BPDE in a dose-dependent manner.

**Conclusions:**

Our results suggest that iAs^III^ enhanced the formation of BPDE–DNA adducts by increasing the cellular uptake of BPDE. Therefore, the ability of arsenic to increase the bioavailability of other carcinogens may contribute to arsenic co-carcinogenicity.

Inorganic arsenic compounds have been shown to enhance the mutagenicity induced by ultraviolet (UV) light, benzo[*a*]pyrene (B[*a*]P), X rays, alkylating agents, and DNA cross-linking compounds in cultured mammalian cells (Pott et al. 2001; Rossman 2003; Styblo et al. 2002). Arsenic may modulate the efficiency of carcinogen–adduct formation, the repair efficiency of carcinogen adducts, or the fidelity of translesion synthesis. Currently, the data are scant regarding the effects of arsenic on the formation of carcinogen–DNA adducts. Only a few studies investigated the effects of arsenic on the formation of DNA adducts induced by B[*a*]P or its reactive metabolite B[*a*]P diol epoxide (BPDE) with conflicting results (Evans et al. 2004; Fischer et al. 2005; Maier et al. 2002; Schwerdtle et al. 2003; Tran et al. 2002). Different methylation patterns of inorganic arsenic in the cell or tissue systems used in those previous studies may partly lead to this variability.

Maier et al. (2002) showed that exposure of mouse hepatoma Hepa-1 cells to low concentrations of arsenite (iAs^III^) increased B[*a*]P-induced BPDE–DNA adduct levels by as much as 18-fold, whereas iAs^III^ treatment did not alter the adduct removal kinetics. An *in vivo* study from the same research group reported that iAs^III^ co-treatment increased the average BPDE–DNA adduct levels in both mouse lungs and skin, with the increase (~ 2-fold) in the lungs being statistically significant (*p* = 0.038) (Evans et al. 2004). Fischer et al. (2005), in another *in vivo* study using mice, found no additional stable BPDE–DNA adducts in the group exposed to iAs^III^ plus B[*a*]P compared with the group exposed to B[*a*]P alone. However, in a study with Sprague-Dawley rats, Tran et al. (2002) observed that iAs^III^ decreased B[*a*]P-induced BPDE–DNA adduct formation. The ^32^P-post-labeling assay was used to detect the DNA adducts in these four studies. Recently, Schwerdtle et al. (2003) examined five arsenic species—iAs^III^, monomethylarsonous acid (MMA^III^), dimethylarsinous acid (DMA^III^), monomethylarsonic acid (MMA), and dimethylarsinic acid (DMA^V^)—for their effects on the formation and repair of BPDE–DNA adducts in A549 human lung cancer cells. By using a high-performance liquid chromatography (HPLC)/fluorescence assay, they observed that iAs^III^ and MMA^III^ increased the BPDE–DNA adduct formation in the concentration range being investigated. In this study iAs^III^ was found to start to enhance adduct formation at 25 μM and achieved 40% more adducts at 75 μM. Unfortunately, no explanations were offered for the increased formation of BPDE–DNA adducts.

One limitation of the previous studies is that DNA repair-proficient systems were used. Therefore, the effects of arsenic on adduct repair could not be differentiated from its effects on adduct formation. In other words, the apparent formation enhancement might simply result from repair inhibition within the co-incubation period included in the treatment protocols of the previous studies. To clarify these issues, we use a nucleotide excision repair (NER)-deficient SV40-transformed *Xeroderma pigementosum* complementation group A (XPA) fibroblast (GM04312C) cell line so that changes in adduct levels can be unambiguously attributed to the effects of arsenic on adduct formation. NER is responsible for removing bulky lesions such as BPDE–DNA adducts from the genome. Abrogation of the p53 function by SV40 transformation could further diminish DNA repair (Bowman et al. 2000). In the present study BPDE was used to generate BPDE–DNA adducts. Thus, problems associated with the metabolic pathways—for example, the phase I bioactivation of B[*a*]P—were avoided. We also examined the influence of iAs^III^ on the glutathione (GSH) system and on chromatin structure, both of which have the potential to modulate BPDE–DNA adduct levels.

## Materials and Methods

### Chemicals

Racemic *anti*-BPDE and [^3^H]-*anti*-BPDE were supplied by the NCI Chemical Carcinogen Repository (Midwest Research Institute, Kansas City, MO). To avoid hydrolysis of the epoxide, we always prepared a stock solution of BPDE fresh by dissolving BPDE in anhydrous tetrahydrofuran (THF) (> 99.9% purity; Sigma-Aldrich, St. Louis, MO) immediately before use. iAs^III^ was obtained as an arsenic atomic absorption standard solution from Aldrich (Milwaukee, WI) and used as a stock solution with a concentration of 13.3 mM.

### Cells and cell cultures

SV40 transformed XPA fibroblast GM04312C cells and human normal fibroblast GM00038B cells were obtained from the NIGMS (National Institute of General Medical Sciences) Human Genetics Cell Repository (Camden, NJ). Human normal fibroblast CRL2522 cells were obtained from the American Type Culture Collection (Rockville, MD). Cells were cultivated in Dulbecco’s modified Eagle’s medium/nutrient mixture F-12 (DMEM/F12) (1:1 ratio) (Gibco BRL, Rockville, MD) supplemented with 10% fetal bovine serum, penicillin (50 U/mL), streptomycin (50 mg/mL), l-glutamine (2 mM), nonessential amino acid (0.1 mM), and sodium pyruvate (1 mM). The cells were seeded in 100-mm dishes at a density of 1 × 10^6^ cells per dish and maintained at 37°C in humidified air containing 5% CO_2_. The cells were grown to about 80–90% confluence for treatments unless otherwise stated.

### Treatment of cells

We pretreated GM04312C cells with iAs^III^ at the indicated concentrations for the respective experiments in complete growth medium for 24 hr. Cells were washed twice with phosphate-buffered saline (PBS) before the addition of BPDE in serum-free medium. The final concentration of BPDE was 0.5 μM. After a 30-min incubation, BPDE was removed, and the cells were washed with PBS 3 times, and their DNA was extracted for measurement of the BPDE–DNA adducts. The concentrations of THF did not exceed 0.001% to avoid the influence of THF on the cells.

### Neutral red uptake and colony-forming assays

We determined the early cytotoxic response of GM04312C cells to iAs^III^ by measuring the ability of exposed cells to incorporate neutral red (NR) into lysosomes. Cells were seeded at densities approximating 2,500 cells per well in a 96-well plate and allowed to grow for 3 days. After treatment with iAs^III^ at various concentrations for 24 hr, cells were washed twice with PBS and assayed for NR uptake using a TOX-4 kit per the manufacturer’s instruction (Sigma). Briefly, cells were exposed to NR in growth media for 2 hr and rinsed with PBS before the NR dye was released. Absorbance was measured at 540 nm with background subtraction at 690 nm, using a SPECTRA MAX 190 microplate spectrophotometer controlled by SOFTmax PRO 4.0 (Molecular Devices Corp., Sunnyvale, CA).

For the colony-forming assay, exponentially growing GM04312C cells were trypsinized and resuspended in DMEM/F12 medium. Cells were seeded into 60-mm dishes at densities from 300 to 2,500 cells per dish and allowed to attach overnight so that no visible cell detachment was observed during treatment. Cells were treated with iAs^III^ at various concentrations for 24 hr. After treatment, cells were washed 3 times with ice-cold PBS and restored in fresh growth medium for colony formation. After 2–3 weeks, colonies were stained with 0.25% methylene blue and counted, and the cloning capability was compared with the plating efficiency of untreated control cells.

### DNA isolation

Cells were lysed in DNAzol reagent (Invitrogen Life Technologies, Carlsbad, CA), and genomic DNA was precipitated with ice-cold 99.9% ethanol and washed twice with cold 70% ethanol. The DNA pellet was air-dried and resuspended in deionized water and the solution was placed in an incubator at 37°C overnight to facilitate the redissolution of DNA. DNA concentrations were measured at 260 nm using a SmartSpec 3000 spectrometer (Bio-Rad Laboratories, Cambridge, MA).

### Detection of BPDE–DNA adducts

In the present study we used a capillary electrophoresis laser-induced fluorescence (CE-LIF)-based immunoassay, as described previously (Le et al. 1998; Wang et al. 2003), to detect BPDE–DNA adducts. Typically, 1 μg of DNA was heat-denatured at 100°C for 5 min followed by cooling on ice for 3 min. Denatured DNA was incubated with a mouse *anti*-BPDE antibody (Clone 8E11, isotype IgG_1_;Trevigen Inc., Gaithersburg, MD) and a goat *anti*-mouse antibody provided in a Zenon Alexa Fluor 546 mouse IgG_1_-labeling kit (Molecular Probes, Eugene, OR). We used an incubation buffer (10 mM Tris and 80 mM glycine adjusted with acetic acid to pH 7.8) to bring the total sample volume to 20 μL. After overnight incubation on ice in the dark, samples (~ 10 nL) were electrokinetically injected into the capillary using an injection voltage of 10 kV for 10 sec. The separation was carried out at room temperature with a separation voltage of 20 kV. The running buffer was a Tris–glycine mixture containing 30 mM Tris and 170 mM glycine, at pH 8.3. Between runs, the capillary was rinsed electrophorectically for 5 min with 0.02 M NaOH and 5 min with the running buffer.

### Cellular glutathione content

Cellular GSH content was determined using a Bioxytech GSH-400 colorimetric assay kit (Oxis International, Portland, OR). Cells (10^6^–10^7^) were trypsinized, centrifuged, and washed with PBS. They were then resuspended in 100 μL of ice-cold metaphosphoric acid. After four cycles of freeze–thaw, the solution was centrifuged at 10,000 × *g* at 4°C for 10 min. The clear supernatant was collected at 4°C for the subsequent assay. Reagent R1 and NaOH from the assay kit were added to the supernatant. After incubation at 25°C for 10 min in the dark, the absorbance of the solution was measured at 400 nm. The GSH concentration in the solution was calculated from the absorbance and a prestored calibration curve of a GSH standard. The cellular GSH content is expressed as nmoles of GSH per million cells.

### *Glutathione* S*-transferase activity assay.*

Cellular glutathione *S*-transferase (GST) activity was measured by a GST colorimetric activity assay kit (Biovision, Cedarlane Laboratory Ltd., Mountain View, CA) using 1-chloro-2,4-dinitrobenzene (CDNB) as the substrate. Briefly, the cells were trypsinized, centrifuged, and homogenized in 100 μL of GST sample buffer. After centrifugation at 10,000 × *g* at 4°C for 10 min, the supernatant was collected for the subsequent assay. GSH and CDNB in GST assay buffer were added to the supernatant and mixed. Absorbance was read at 340 nm using a SPECTRA MAX 190 microplate spectrophotometer controlled by SOFTmax PRO 4.0 (Molecular Devices Corp.). Absorbance readings were taken repeatedly for a minimum of five time intervals to obtain enzyme kinetic information. GST activity was expressed as nmoles of CDNB reduced per minute per million cells.

### Chromatin accessibility

A denaturation sensitivity assay was used to examine chromatin accessibility, as described by Rubbi and Milner (2003), with some modifications. Cells were cultured in DMEM/F12 (1:1 ratio) (Gibco BRL) supplemented with 10% fetal bovine serum on coverslips placed in 35-mm dishes. After reaching 80–90% confluence, the cells were incubated with iAs^III^ at various concentrations. After a 24-hr incubation, the cells were washed and fixed with 2% paraformaldehyde for 30 min, then incubated with 50 μg/mL RNase A (Sigma) in PBS at room temperature for 1 hr. The cells were then denatured for 30 sec with 0.1 M HCl. The denaturation was stopped with 20 μg/mL acridine orange (AO) (Molecular Probes) in 0.1 M phosphate/citrate buffer (pH 2.6), and the coverslips were mounted with the addition of 1,4-diazobicyclo-[2,2,2]-octane (DABCO, #D2522; Sigma) and Mowiol 488 (#475904; Calbiochem, La Jolla, CA). A Zeiss LSM510 laser scanning confocal microscope (Carl Zeiss, Jena, Germany) with an F-Fluar 40×, NA 1.3 objective lens was used to obtain fluorescence images. The cell samples were scanned using a 488-nm argon ion laser for excitation (0.75% transmission); both green (dsDNA) and red (ssDNA) fluorescence signals were detected through the 505/50BP and 650LP filters, respectively. The fraction of dsDNA was calculated as *F_dsDNA_* = *G*/(*G+R*) and displayed numerically.

### Cellular uptake of BPDE

Eighty to ninety percent confluent GM04312C cells grown in 6-well plates were treated with iAs^III^ for 24 hr. After being washed twice with PBS, the cells were incubated with 0.5 μM [^3^H]-*anti*-BPDE (2030 mCi/mmol) (Chemsyn Science Laboratories, Lenexa, KS) for 30 min. After washing with PBS 3 times, the cells were lysed in 0.2 M NaOH before radioactivity measurement. Cells in parallel wells were trypsinized and counted. The radioactivity of each sample was determined with an LS5801 liquid scintillation counter (Beckman Coulter, Fullerton, CA) and expressed as cpm/10^6^ cells.

## Results

### Repair capability of the chosen XPA cell line

**Figure 1:**
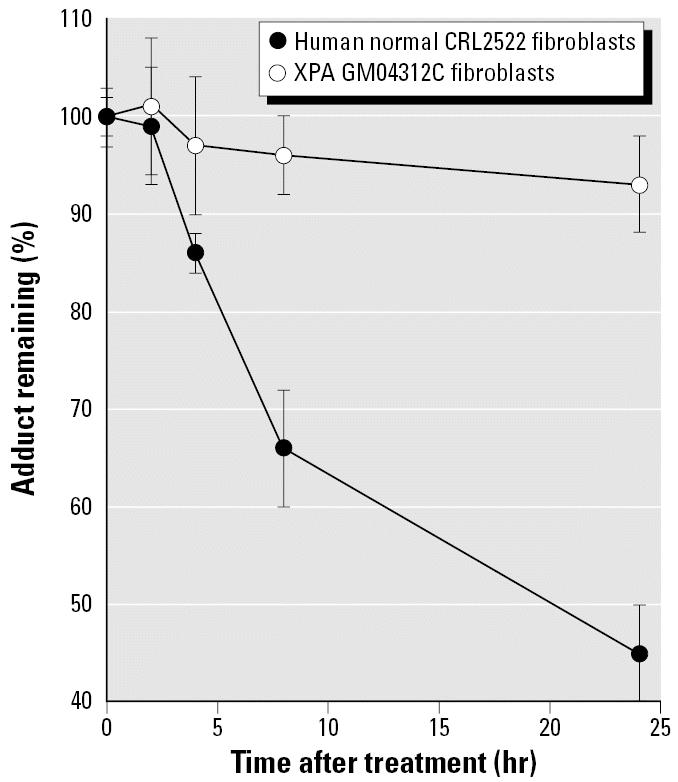
Repair of BPDE–DNA adducts in human normal CRL2522 fibroblasts and XPA GM04312C fibroblasts. More than 90% confluent cells were treated with 1 μM BPDE for 30 min, then allowed to repair in complete medium for 0, 2, 4, 8, or 24 hr. Error bars indicate 1 SD of four determinations from duplicate experiments of cell treatment.

To justify the use of GM04312C cells as NER-deficient cells for studying the effects of arsenic on the formation of BPDE–DNA adducts, repair experiments with GM04312C and CRL2522 (repair proficient, positive control) cells were carried out. The repair kinetics in these cell lines are shown in Figure 1. Ninety percent confluent cells were used to limit posttreatment replication. While 55% of BPDE–DNA adducts were removed within 24-hr after BPDE treatment in normal fibroblast CRL2522 cells, the differences in BPDE–DNA adduct levels in the GM04312C cells between different repair times up to 24 hr were not statistically significant (*p* > 0.05). These results confirmed that GM04312C cells lack the ability to repair BPDE–DNA adducts.

### Cytotoxicity of iAs^III^ to GM04312C cells

**Figure 2:**
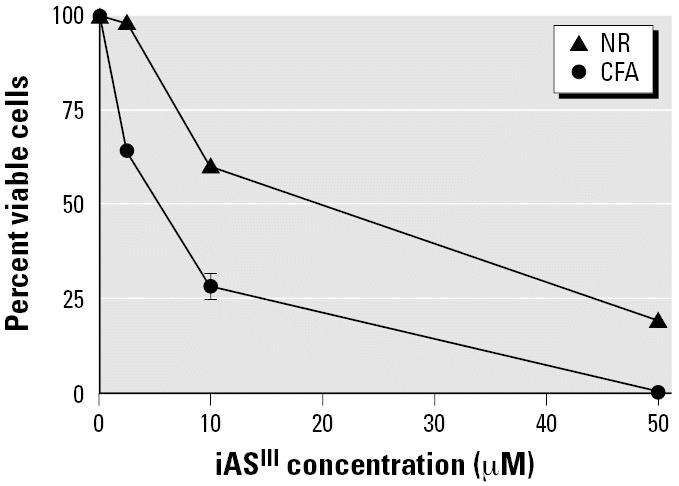
Effects of iAs^III^ on the cell viability of GM04312C cells examined by neutral red (NR) uptake and a colony-forming assay (CFA). For NR experiments, approximately 2,500 cells were seeded in each well of a 96-well plate and allowed to grow for 3 days. The cells were treated with iAs^III^ for 24 hr, and the NR uptake ability was examined. For CFA experiments an appropriate number of cells (300–2,500) were seeded in each designated dish and allowed to attach overnight. After a 24-hr treatment with iAs^III^ at the indicated concentrations, cells were restored in fresh growth medium for colony formation. Error bars indicate the SE of four determinations from two experiments.

The cytotoxicity of iAs^III^ was determined both by the NR uptake assay immediately after exposure to arsenic and by the colony-forming assay, which requires cell growth for 2–3 weeks after exposure (Figure 2). When we used the NR uptake assay, the cytotoxic effects were observed only in cells exposed to iAs^III^ at concentrations > 2.5 μM. After treatment with 10 μM iAs^III^, 60% of the cells remained viable, whereas treatment with 50 μM iAs^III^ left 20% of cells viable. Colony-forming ability, however, was inhibited at all concentrations of iAs^III^ tested (2.5–50 μM) in a dose-dependent manner. Only 28% of the cells exposed to 10 μM iAs^III^ were able to form colonies. No colonies were observed at 50 μM iAs^III^. The colony-forming assay is known to be a very sensitive method of toxicity evaluation, but it may underestimate early cell survival (Hamdan et al. 1999).

### Effects of iAs^III^ on the formation of BPDE–DNA adducts

**Figure 3:**
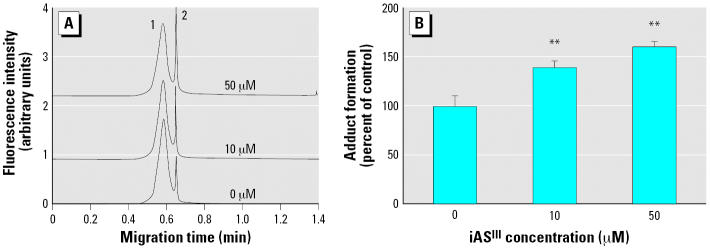
Effects of iAs^III^ on the formation of BPDE–DNA adducts. GM04312C cells were pretreated with iAs^III^ at the indicated concentrations for 24 hr, then incubated with 0.5 μM BPDE for 30 min. The cells were lysed, and DNA was extracted for analysis of BPDE–DNA adducts using a CE-LIF–based immunoassay. (*A*) Electropherograms: peak 2 corresponds to the antibody complex with the BPDE-damaged DNA; peak 1 corresponds to the excess antibodies. (*B*) Relative adduct levels compared to those of controls. Adduct levels obtained from BPDE incubation only were normalized to 100%. **Statistically significant difference from controls (*p* < 0.010) using one-way Student’s *t*-test. Error bars indicate 1 SD from three experiments.

The GM04312C cells were treated with graded concentrations (0–50 μM) of iAs^III^ for 24 hr. After a 30-min incubation with 0.5 μM BPDE, the BPDE–DNA adducts in the cellular DNA were measured by the CE-LIF–based immunoassay. Figure 3 shows that iAs^III^ increased adduct formation by 39% at 10 μM and by 60% at 50 μM.

### Effects of iAs^III^ on GSH-mediated inactivation of BPDE

**Figure 4:**
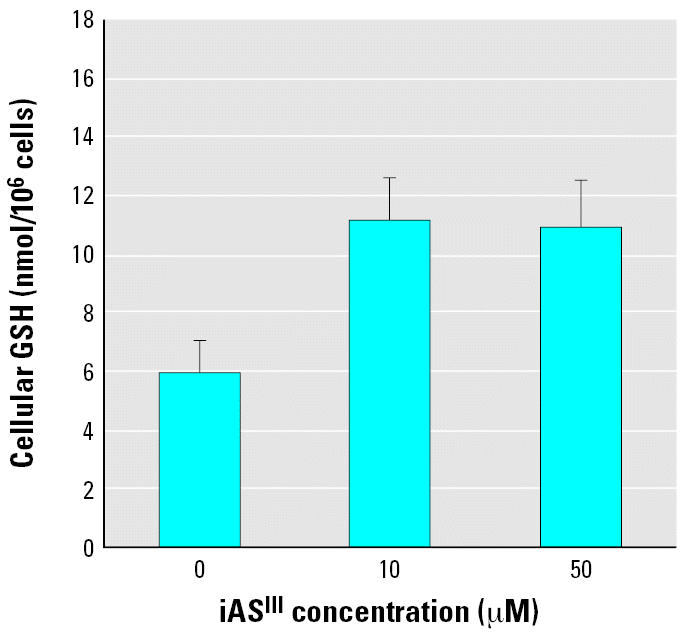
Effects of iAs^III^ on cellular-reduced GSH levels. Eighty to ninety percent confluent GM04312C cells were treated with iAs^III^ at the concentrations indicated for 24 hr, then homogenized for GSH detection. Error bars indicate 1 SD from three experiments.

The GSH levels in GM04312C cells increased significantly from 5.8 nmol/10^6^ cells to 11 nmol/10^6^ cells after a 24-hr treatment with 10 μM iAs^III^. A higher concentration (50 μM) of iAs^III^ did not lead to a further increase in the GSH levels (Figure 4).

**Figure 5:**
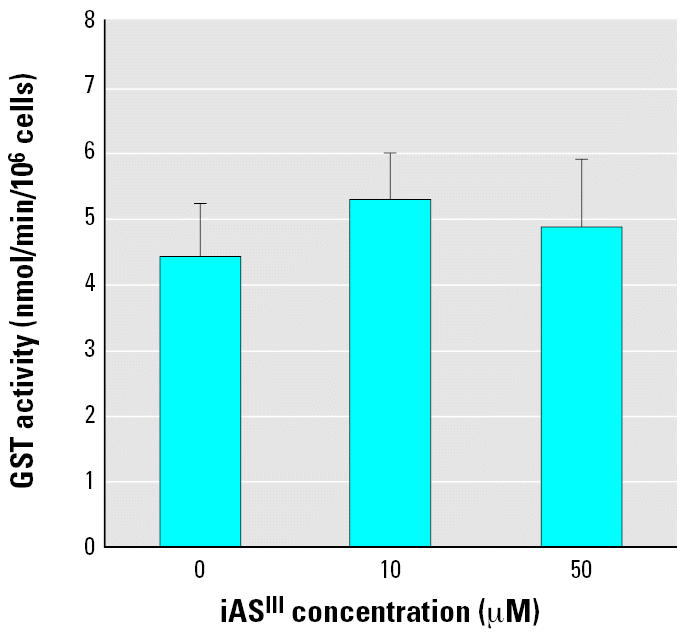
Effects of iAs^III^ on the activity of GST. Eighty to ninety percent confluent GM04312C cells were treated with iAs^III^ at the concentrations indicated for 24 hr, then homogenized for the GST activity assay. Error bars indicate 1 SD from three experiments.

We also measured GST activity of the GM04312C cells after pretreatment with iAs^III^ for 24 hr, as shown in Figure 5. Pretreatment with iAs^III^ had no significant effect on GST activity in the concentration range under investigation.

### Effects of iAs^III^ on chromatin accessibility

**Figure 6:**
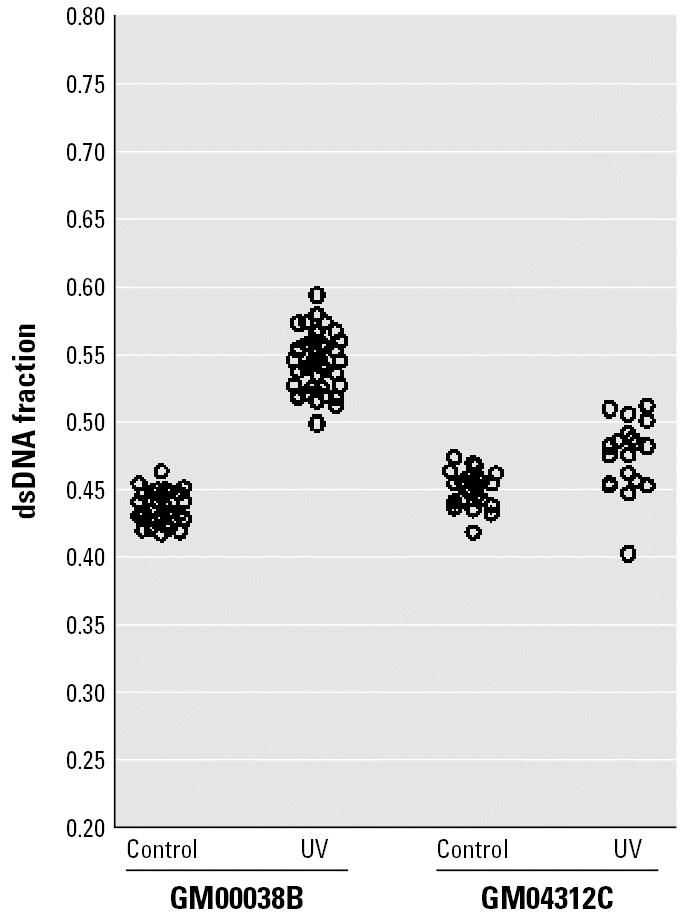
DNA denaturation sensitivity of exponentially growing GM00038B and GM04312C cells with or without UVC irradiation. Cells were irradiated with UVC at 4 J/m^2^ and incubated for 1 hr in culture medium, then subjected to the HCl/AO assay. At least three sampling areas were pooled, and each circle represents a single cell.

A confocal microscope-based technique originally devised by Dobrucki and Darzynkiewicz (2001) to examine chromatin relaxation *in situ* was adapted in our study to investigate the effect of iAs^III^ on chromatin accessibility in GM04312C cells. After removal of RNA and partial denaturation of DNA by HCl, AO was used to stain nondenatured dsDNA and denatured ssDNA, resulting in green and red fluorescence, respectively. Green fluorescence is associated with relaxed chromatin and red fluorescence with condensed chromatin. As a method-positive control, GM00038B cells had relaxed chromatin structure after 4 J/m^2^ UVC irradiation, which is in agreement with a previous report (Rubbi and Milner 2003). Conversely, the same UVC dose did not lead to a change in chromatin structure in GM04312C cells (Figure 6). Attenuation of p53 functionality by SV40 transformation might be the cause for the difference because functional p53 was demonstrated to be required for UV-induced chromatin relaxation (Rubbi and Milner 2003).

**Figure 7:**
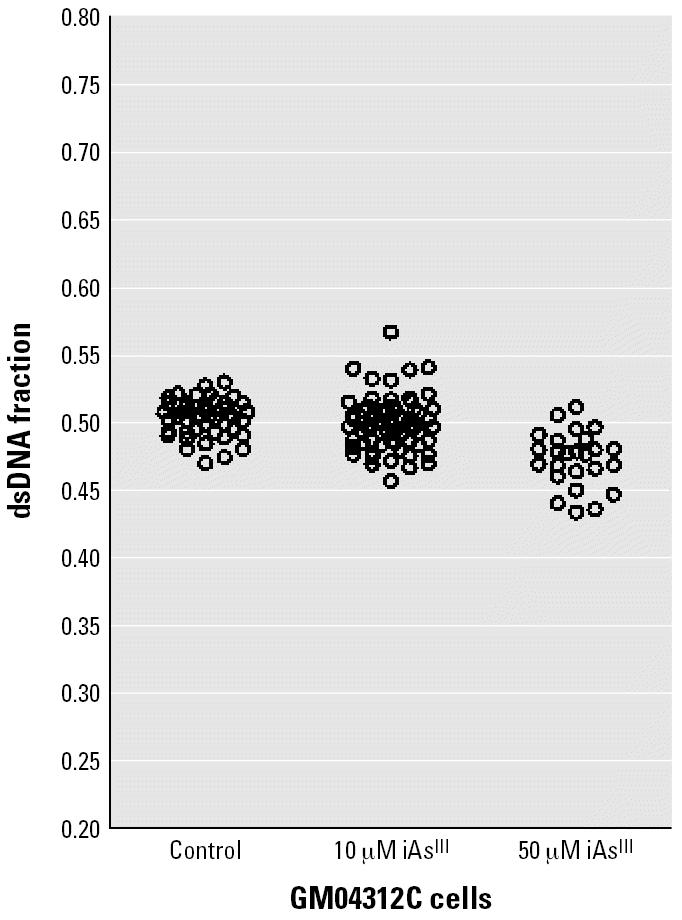
DNA denaturation sensitivity measured by the HCl/AO assay on 80–90% confluent GM04312C cells after treatment with iAs^III^ for 24 hr. At least three sampling areas were pooled, and each circle represents a single cell.

Pretreatment of GM4312C cells with iAs^III^ at concentrations up to 50 μM also failed to relax the chromatin structure (Figure 7). In fact, cells treated with this dose appeared to have more condensed chromatin than the controls.

### Effects of iAs^III^ on the cellular uptake of BPDE

**Figure 8:**
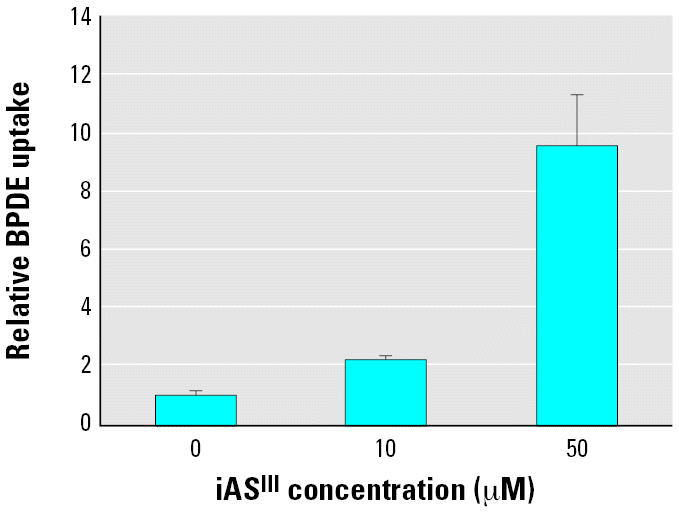
iAs^III^ increased the cellular uptake of BPDE in a concentration-dependent manner. The GM04312C cells were pretreated with iAs^III^ at the indicated concentrations, then incubated with 0.5 μM [^3^H]-BPDE for 30 min. After cell lysis, radioactivity was measured and normalized against the control. Error bars indicate 1 SD of four determinations from two experiments.

Radioactive [^3^H]-BPDE was used to probe the effect of iAs^III^ on the cellular uptake of BPDE after iAs^III^ pretreatment. The GM04312C cells exposed to 0.5 μM [^3^H]-BPDE in the absence of iAs^III^ resulted in a radioactivity measure of 55,000 cpm/10^6^ cells, which corresponded to approximately 12.3 pmol of BPDE in 10^6^ cells. Treatment with iAs^III^ led to increased cellular uptake in a concentration-dependent manner (Figure 8). At 10 μM iAs^III^ the uptake of BPDE increased by 1.2-fold, whereas at 50 μM iAs^III^ the uptake increased by 8.6-fold.

## Discussion

Resistance to arsenic in mammalian cells has been shown to correlate with high levels of intracellular GSH and high GST activity (Brambila et al. 2002; Lee et al. 1989; Lo et al. 1992). Depletion of GSH has been shown to block arsenic methylation (Buchet and Lauwerys 1987) and results in increased cytotoxicity and clastogenicity of arsenic (Oya-Ohta et al. 1996). GST-catalyzed GSH conjugation of BPDE is believed to be the most important enzymatic pathway to inactivate BPDE (Hu et al. 1996; Robertson et al. 1986a, 1986b). The results of our experiments indicated that iAs^III^ increased GSH levels in a concentration-dependent manner (Figure 4) and had no significant effect on GST activity (Figure 5). However, BPDE–DNA adducts increased after iAs^III^ pretreatment (Figure 3). This lack of correlation is in agreement with the observation by Maier et al. (2002). They treated cells with iAs^III^ for 30 min before addition of B[*a*]P and determined the GSH levels after 1.5 hr of iAs^III^ and B[*a*]P co-treatment. They either depleted GSH with l-buthionine-*S,R*-sulfoximine or replenished GSH with glutathione ethylester to modulate cellular GSH levels. The changes in the cellular GSH status led to noticeable effects on the yields of the B[*a*]P-induced BPDE–DNA adducts. However, those changes could not be achieved by micromolar iAs^III^ treatment alone because of the millimolar levels of cellular GSH. Their results did not suggest that iAs^III^ increases BPDE–DNA adducts through direct competition with B[*a*]P metabolites for the cellular GSH pool.

Chromatin relaxation carries an increased risk of certain types of DNA damage. Evidence has shown that compact chromatin is protective against DNA double-strand breaks and oxidative DNA damage. This protection is reduced after chromatin decondensation (Ljungman and Hanawalt 1992). UV-induced global chromatin relaxation rendered DNA more susceptible to a number of DNA-damaging agents (Ljungman 1989). To test if enhanced formation of BPDE–DNA adducts was due to a more relaxed chromatin structure, we examined chromatin accessibility in GM04312C cells after iAs^III^ treatment. Our study showed that iAs^III^ treatment did not lead to more relaxed chromatin (Figure 7), which means that BPDE forms DNA adducts regardless of additional chromatin relaxation. In line with our results, no differences in the BPDE–DNA adduct levels were shown across different cell cycle phases (Shinozaki et al. 1998), although it is known that changes in chromatin structure are associated with different cell cycle phases. Similarly, the BPDE–DNA adduct levels in quiescent cells were close to the levels in rapidly proliferating cells (Cunningham et al. 1989). Furthermore, histone hyperacetylation by butyrate treatment did not influence the initial levels of BPDE–DNA adducts nor did it change the rate of removal of BPDE–DNA adducts from chromatin in either normal human fibroblasts or XP fibroblasts (Kootstra 1982). The author concluded that the subtle changes in chromatin brought about by histone acetylation had no influence on these processes.

The attempt to correlate cellular GSH levels, GST activity, or chromatin accessibility with BPDE–DNA adduct levels is driven and justified by the assumption that cellular bioavailability of BPDE remained unchanged after iAs^III^ treatment. However, iAs^III^ might have enhanced the formation of BPDE–DNA adducts simply by increasing the stability of BPDE in aqueous medium or the cellular uptake of BPDE. In our treatment protocols, the cells were washed following iAs^III^ treatment and then incubated with BPDE for 30 min. The pretreatment of iAs^III^ was not expected to affect BPDE stability in aqueous medium. However, as shown in Figure 8, the uptake of BPDE was clearly affected by iAs^III^ pretreatment. This increased uptake of BPDE was in parallel with the enhanced formation of BPDE–DNA adducts after iAs^III^ treatment. However, the increased uptake of BPDE did not translate into direct proportional enhancement of BPDE–DNA adduct formation (Figures 3 and 8), i.e., 50 μM iAs^III^ increased the uptake of BPDE by almost 10-fold, whereas adduct formation increased by only 1.6-fold. iAs^III^ may decrease BPDE efflux by inhibiting the activity of GSH-conjugate transporters. There is a positive correlation between the intracellular accumulation of the GSH conjugate of BPDE and increased formation of BPDE–DNA adducts in cells lacking multi-drug resistance-associated protein 2 (MRP2) (Srivastava et al. 2002). Most evidence to date indicates that iAs^III^ induces expression of multi-drug resistance transport proteins (Chin et al. 1990; Kioka et al. 1992; Kojima et al. 2006; Takeshita et al. 2003; Vernhet et al. 2001). Because the transport of the GSH conjugate of BPDE is adenosine triphosphate (ATP)-dependent (Srivastava et al. 1998), the possibility of ATP depletion by iAs^III^ should be considered (Yih et al. 1991). Recently, iAs^III^ has been shown to modulate the induction of some detoxifying phase II genes mediated by the aryl hydrocarbon receptor such as quinone oxido-reductase (QOR) (Elbekai and El-Kadi 2004; Kann et al. 2005). However, it is unlikely for QOR to inactivate BPDE because BPDE is a poor substrate (Iskander et al. 2005; Thakker et al. 1977). Despite this, after entry into the cell, BPDE may be sequestered or inactivated by alternative minor pathways (Dock et al. 1986; Yang and Gelboin 1976), which may be affected by arsenic.

Currently, the data on the effects of arsenic on the uptake of other chemicals in human cells are limited. Ochi (1997) observed that micromolar concentrations of iAs^III^ increased the uptake of cystine in Chinese hamster V79 cells. It was suggested that the cystine transport system was induced by iAs^III^ treatment. Similarly, micromolar concentrations of iAs^III^ were shown to increase cystine uptake in AG06 keratinocytes (Schuliga et al. 2002). In the same cell line, low doses of iAs^III^ (< 1 μM) were also shown to increase NR dye uptake, whereas higher doses of iAs^III^ up to 3μM decreased the dye uptake (Snow et al. 1999). In 3T3-L1 adipocytes, iAs^III^ inhibited glucose uptake by interfering with a glucose transporter (Walton et al. 2004). For BPDE, no transporters have been reported for its cellular uptake. However, the log*K*_ow_ (a measure of a chemical’s partition between octanol and water phases) of BPDE may be estimated as 2.5–3.5 on the basis of its SMILES (simplified molecular input line entry system) notation using ALOGPS 2.1 software (free software available at www.vcclab.org/lab/alogps) compared with the log*K*_ow_ of 6.3 for its parent compound B[*a*]P (more hydrophobic). Therefore, although B[*a*]P enters cells by passive diffusion (Plant et al. 1985; Brunette and Katz 1975), the possibility that BPDE is taken up by active as well as by passive transport must be considered. Pretreatment with iAs^III^ may have a negative impact on the active transport while the passive transport is favored. In the latter case, iAs^III^ may enhance BPDE uptake by binding to sulfhydryl groups in membrane proteins and thus prevent adsorption of BPDE to the cell membrane during BPDE influx.

## Conclusions

Pretreatment with subtoxic concentrations of iAs^III^ enhanced the formation of BPDE–DNA adducts in a DNA repair-deficient human cell line. Treatment with iAs^III^ had no significant effect on the GSH conjugation system, the major inactivation pathway for BPDE nor did it affect chromatin structure. However, iAs^III^ increased the cellular uptake of BPDE, which paralleled the elevated levels of BPDE–DNA adducts. Our results suggest that modulation of cellular uptake of BPDE by iAs^III^ may be a major if not the determining factor leading to the observed enhancement of BPDE–DNA adduct formation. It should be noted that the concentration of 10 μM iAs^III^ used in our study is relevant to some real-world exposure scenarios and in biological systems (National Research Council 2001; Snow et al. 2005). Our study implies that the issue of bioavailability should be considered when co-exposure to more than one carcinogen is examined for co-carcinogenicity. BPDE is a reactive metabolite of benzo[*a*]pyrene, a common environmental carcinogen. iAs^III^ may contribute to its co-carcinogenicity with BPDE and other relatively polar environmental pro-carcinogens that have cellular uptake mechanisms similar to those of BPDE, aside from its well-demonstrated inhibitory effect on DNA repair.
